# DNA methylome, R-loop and clinical exome profiling of patients with sporadic amyotrophic lateral sclerosis

**DOI:** 10.1038/s41597-024-02985-y

**Published:** 2024-01-24

**Authors:** Orsolya Feró, Dóra Varga, Éva Nagy, Zsolt Karányi, Éva Sipos, József Engelhardt, Nóra Török, István Balogh, Borbála Vető, István Likó, Ábel Fóthi, Zoltán Szabó, Gábor Halmos, László Vécsei, Tamás Arányi, Lóránt Székvölgyi

**Affiliations:** 1 MTA-DE Momentum, Genome Architecture and Recombination Research Group, Department of Molecular and Nanopharmaceutics, Faculty of Pharmacy, University of Debrecen, H-4032 Debrecen, Hungary; 2 Doctoral School of Pharmaceutical Sciences, Faculty of Pharmacy, University of Debrecen, H-4032 Debrecen, Hungary; 3 Department of Internal Medicine, Faculty of Medicine, University of Debrecen, Debrecen, H-4032 Hungary; 4 Department of Biopharmacy, Faculty of Pharmacy, University of Debrecen, H-4032 Debrecen, Hungary; 5 Department of Neurology, Albert Szent-Györgyi Medical School, University of Szeged, Szeged, Hungary; 6 Division of Clinical Genetics, Department of Laboratory Medicine, Faculty of Medicine, University of Debrecen, H-4032 Debrecen, Hungary; 7 Institute of Enzymology, Research Centre for Natural Sciences, Budapest, Hungary; 8 Department of Emergency Medicine, Faculty of Medicine, University of Debrecen, H-4032 Debrecen, Hungary; 9 Department of Molecular Biology, Semmelweis University, Budapest, Hungary; 10 Department of Biochemistry and Molecular Biology, Faculty of Medicine, University of Debrecen, Debrecen, H-4032 Hungary

**Keywords:** Amyotrophic lateral sclerosis, Epigenomics

## Abstract

Amyotrophic lateral sclerosis (ALS) is a fatal neurodegenerative disorder characterized by the death of motor neurons, the aetiology of which is essentially unknown. Here, we present an integrative epigenomic study in blood samples from seven clinically characterised sporadic ALS patients to elucidate molecular factors associated with the disease. We used clinical exome sequencing (CES) to study DNA variants, DNA-RNA hybrid immunoprecipitation sequencing (DRIP-seq) to assess R-loop distribution, and reduced representation bisulfite sequencing (RRBS) to examine DNA methylation changes. The above datasets were combined to create a comprehensive repository of genetic and epigenetic changes associated with the ALS cases studied. This repository is well-suited to unveil new correlations within individual patients and across the entire patient cohort. The molecular attributes described here are expected to guide further mechanistic studies on ALS, shedding light on the underlying genetic causes and facilitating the development of new epigenetic therapies to combat this life-threatening disease.

## Background & Summary

Amyotrophic lateral sclerosis (ALS) is a devastating neurodegenerative disorder that affects the motor neurons in the brain and spinal cord. It is characterised by the progressive degeneration and loss of these motor neurons, leading to a gradual deterioration of muscle control and function. As a result, ALS patients experience muscle weakness, paralysis, and ultimately, difficulty or inability in breathing, swallowing, and speaking. The molecular cause of ALS is complex and not fully understood. Diagnosing the disease is a significant challenge as ALS has similar symptoms to other diseases. Differentiating ALS from these conditions requires a number of medical tests, including genetic testing, electromyography (EMG), as well as MRI scans^1^. Currently, there are no effective proteomic, RNA, or other biomarkers to provide early predictions for this disorder.

ALS is a rare disease with a worldwide incidence of 1.75 cases per 100 000 person per years of follow-up^2^. The familial form (representing 5–10% of cases) is linked to specific gene mutations such as SOD1, C9orf72, TARDBP, and FUS, however, the list of discovered ALS gene mutations is continuously growing. The pathogenesis of sporadic ALS, which constitutes the majority of cases (around 90–95%), is less clear and likely involves a combination of genetic and environmental factors^3–5^. While specific gene mutations are not prevalent in sporadic ALS, genetic variants as risk factors can significantly contribute to disease susceptibility^6^. Pathogenic or likely-pathogenic variants that individually have only modest effects on ALS risk can collectively contribute to an individual’s predisposition (this concept is best supported by evidence in individuals carrying a p.N352S mutation in TARDBP)^7^. Common variants with small effect size and combinations of such variants may also confer genetic risk in sporadic ALS patients, but convincing data demonstrating this are still lacking^8^. Therefore, establishing new genomic data to identify these genetic variants and their combinations in individuals becomes of paramount importance to explain the “missing heritability”^6^ of high-risk (causative) ALS genes. On the other hand, Mendelian inheritance accounts for only a minority of cases, promoting a focus on environmental and epigenomic cues to explain the missing heritability of known ALS mutations^9^. In addition to genetic variants, epigenetic changes such as DNA methylation^10–13^ and R-loops^14–20^ are highly likely to play a role in the development of sporadic ALS. Although the precise pathophysiological mechanisms by which these factors contribute to motor neuron degeneration remain unclear, they may be applicable as diagnostic markers of the disease.

**Fig. 1:**
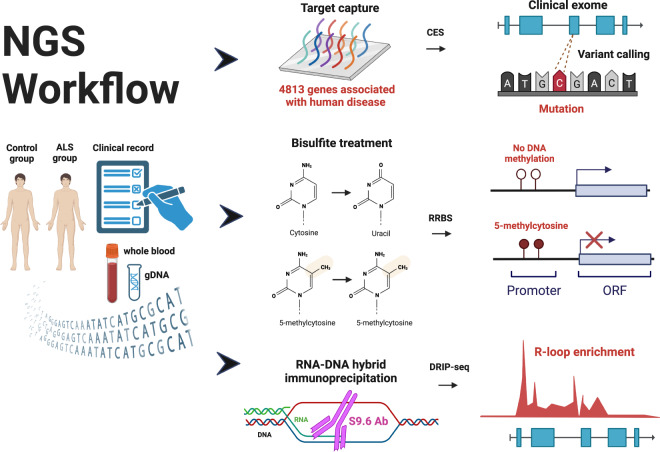
Outline of the next generation sequencing (NGS) experiments. The workflow began with obtaining blood samples from ALS patients with well-defined clinical records and age-matched control individuals. From these blood samples, genomic DNA was extracted that underwent three simultaneous investigations: 1. Clinical exome sequencing (CES) was performed on 4813 genes commonly linked to human diseases. The protein-coding regions (exons) of the target genes were enriched using target capture microarray hybridization prior to NGS. 2. Reduced representation bisulfite sequencing (RRBS) was employed to analyze gene regulatory regions that influence gene transcription through DNA methylation. 3. DNA-RNA hybrid immunoprecipitation sequencing (DRIP-seq) was utilized to capture chromosomal R-loops associated with genic and intergenic regions.

Here, we present a comprehensive epigenomic investigation in blood samples from seven sporadic ALS patients with well-defined clinical characteristics to uncover molecular factors linked to the disease. Our study employed three main techniques: clinical exome sequencing (CES)^21^ to analyze DNA variants, DNA-RNA hybrid immunoprecipitation sequencing (DRIP-seq)^22^ to investigate R-loop accumulation, and reduced representation bisulfite sequencing (RRBS)^23^ to explore DNA methylation levels (Fig. 1). By combining these datasets, we have compiled a compendium of genomic alterations associated with the studied ALS cases.

## Methods

### Patient selection

The clinical diagnosis of ALS cases fulfilled the ‘El Escorial revisited’ clinically definite diagnostic certainty and the complementary ‘Awaji’ criteria for amyotrophic lateral sclerosis^24,25^. All selected patients are considered to have sporadic ALS, as the disease did not occur in their families (Table 1). We confirm that informed consent was obtained from all subjects and that our experiments were approved by the Medical Research Council (Ministry of Interior, Hungary) in accordance with all relevant ethical and legal requirements (approval numbers: 47066-3/2013/EKU(556/2013); 11920-2/2017/EÜIG; 12702-5/2018/EÜIG). We confirm that the ethics agreement allows for the open publication of anonymized genetic data and the participants understood all risks for the open publication of these data, as per informed consent.

**Table 1:** Clinical characteristics of individuals examined in this study.

ID	Group	Sex	Age (at the time of sampling)	Age (at the onset of ALS)	Severity (ALSFRS score*)	Duration of ALS (at the time of sampling; month)	ALS symptoms	Sites of ALS symptoms
ALS61	ALS	female	52	50	38	24	classic (all 3 symptom groups), bulbar, lower and upper limb, pseudobulbar	bulbar: dysarthria, lower MNs > upper MNs
ALS62	ALS	female	54	53	34	12	classic (all 3 symptom groups), bulbar, lower and upper limb	lower extremity weakness /right > left/ mainly lower MNs
ALS69	ALS	male	51	49	43	18	classic (all 3 symptom groups), bulbar, lower and upper limb	bulbar: dysarthria, lower MNs > upper MNs
ALS74	ALS	female	45	45	47	1	lower and upper limb, bulbar not present	bilateral peroneal muscle weakness: difficulty in walking, lower and upper MNs
ALS75	ALS	male	59	57	24	24	lower and upper limb, bulbar not present	lower extremity weakness and clumsiness: difficulty in waking, upper and lower MNs
ALS76	ALS	female	61	60	40	12	lower and upper limb, bulbar not present	slightly asymmetrical extremity weakness: mainly lower MNs
ALS81	ALS	female	69	67	24	18	classic (all 3 symptom groups), bulbar, lower and upper limb	left upper extremity weakness: lower MNs
K039	Ctrl	female	52	N/A	N/A	N/A	N/A	N/A
K107	Ctrl	female	45	N/A	N/A	N/A	N/A	N/A
K128	Ctrl	male	51	N/A	N/A	N/A	N/A	N/A
K161	Ctrl	female	65	N/A	N/A	N/A	N/A	N/A
K190	Ctrl	male	59	N/A	N/A	N/A	N/A	N/A
K257	Ctrl	female	61	N/A	N/A	N/A	N/A	N/A
K485	Ctrl	female	63	N/A	N/A	N/A	N/A	N/A

Five female and two male patients with at least two symptoms of the classic diagnostic hallmarks of ALS were recruited. None of the patients had any other comorbidities. Their median age was 51 years, and the median time from diagnosis to sampling was 15 months. Seven age-matched and gender-matched control individuals were also included in the studies. The severity of the diseases at the time of the onset of ALS patients is based on the ALSFRS^*^ score^41^. The maximal score is 48: not having any signs and symptoms.

### Genomic DNA extraction from whole blood

We prepared aliquots of 1 ml frozen whole blood sample from ALS and control patients and extracted 200 μL of it using the Macherey-Nagel™ NucleoSpin™ Tissue Kit according to the manufacturer’s protocol. In brief, we mixed 200 μL of the whole blood sample with 25 μL of Proteinase K and 200 μL of buffer B3, prewarmed to 65 °C. After vortexing the mixture, we incubated it at 65 °C for 10 minutes. Then, we added 210 μL of ethanol (96 – 100%) to each sample, vortexed again, and loaded the mixture onto a NucleoSpin® Tissue Column placed in a Collection Tube. We centrifuged it for 1 minute at 11,000 × g, followed by washing the silica membrane once with 600 μL of BW buffer and once with 600 μL of B5 buffer. After drying the membrane for five minutes, we eluted the DNA twice with 50 μL of prewarmed (55 °C) elution buffer (5 mM Tris pH 8.5). The same samples were pooled and DNA concentration was measured using a Nanodrop. The samples were then concentrated to 100 μL using a speed vacuum concentrator. Next, the genomic DNA was fragmented by digesting 25ug of DNA in a final volume of 100 μL with HindIII, EcoRI, BsrGI, XbaI, and SspI restriction enzymes at 37 °C for 4 hours. We performed a fragment analysis of 3 μL of the digested sample on a 1% agarose gel stained with GelRed (SigmaAldrich). DNA was cleaned from the enzyme reaction using the Macherey-Nagel™ NucleoSpin™ Gel and PCR Clean-up Kit according to the manufacturer’s protocol, eluting the fragmented DNA with 2 × 50 μL of nuclease-free water.

### Clinical exome seq

**Fig. 2:**
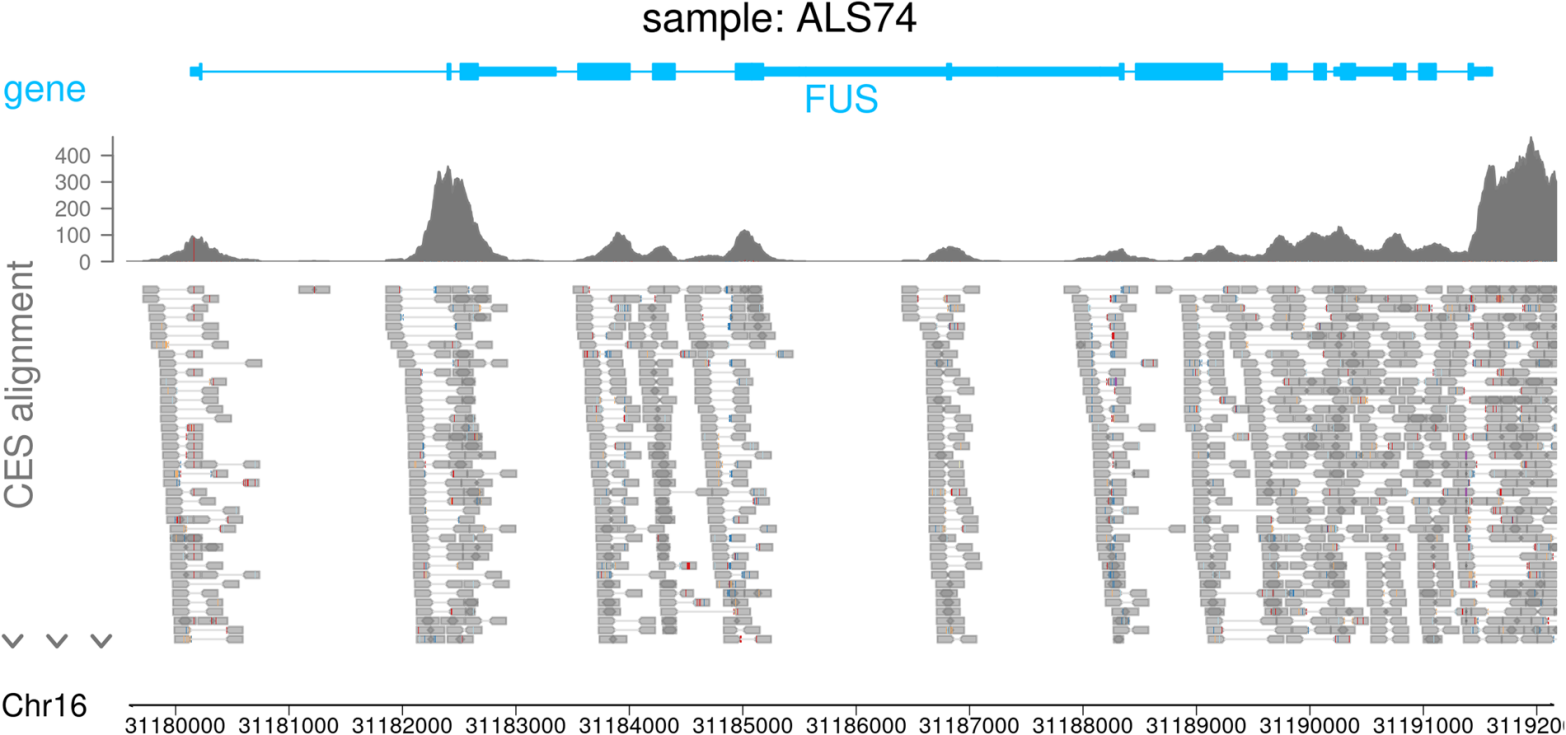
Representative clinical exome sequencing result of an ALS patient. Aligned reads are shown over the FUS gene. The list of variants can be found in the Data Records section.

Clinical exome sequencing was performed by TruSight One Sequencing Panel Kit (Illumina, San Diego, CA) that covers coding regions of 4813 genes associated with human disease. Library preparation was done in accordance with the manufacturer’s instructions. Sequencing was performed on the Illumina MiSeq instrument with the 2 × 150 paired-end sequencing mode. Read alignment and sequence variant analysis were performed using NextGENe Software version 2.4.2.3 (SoftGenetics, State College, PA). The reads were mapped against the human GRCh38 (hg38) reference genome. More than 84% of the target regions were observed with least 20-fold coverage. Variants were recorded when a minimum of 5 reads aligned to the variant position, and the variant was observed in at least 20% of those reads (Fig. 2). Variants were annotated using the gnomAD v2.1.1 (GRCh38 LiftOver) database. The clinical interpretation of variants were extracted from the ClinVar (NCBI) database. Analysis results of the samples were obtained from NextGENe as variant call format (vcf) files. Mutation reports (variant lists) were also exported as text tables (.tsv) and then merged, sorted and formatted using R v4.3.1 https://www.R-project.org). Reference files, databases used in the analyses, and non-default software settings are detailed in the Data Records section.

### Reduced Representation Bisulfite Sequencing (RRBS)

Reduced Representation Bisulfite Sequencing (RRBS) is an established technique for identifying cytosine methylation in genomic regions most relevant to gene regulation. The genomic DNA first undergoes digestion by the MspI restriction enzyme, which recognizes CCGG sites, resulting in genomic fragments starting and ending with a CpG dinucleotide. Since CpGs are unevenly distributed throughout the genome, MspI digestion is followed by size selection that enriches for CpG-rich regions, including CpG islands. These regions are typically located in gene promoters and control gene expression through DNA methylation. We employed the premium RRBS kit of Diagenode to carry out the RRBS protocol including NGS library preparation, as described^26,27^. Briefly, 100 ng of DNA was fragmented by the MspI restriction enzyme, which cleaves DNA without regard to the cytosine methylation state. Following adaptor ligation and size selection by Ampure beads, we pooled up to 6 samples together. These pooled samples underwent bisulfite treatment, purification, and PCR amplification, according to the manufacturer’s recommendations. The final RRBS libraries were quantified using the Qubit dsDNA HS Assay from Life Technologies, and the library’s size distribution profile was assessed using Agilent Bioanalyzer 2100 capillary electrophoresis. Sequencing was performed on an Illumina HiSeq 2500 instrument using 1 × 50 single-end mode. Adapter sequences were removed from the raw reads using Trim Galore v0.6.10 https://github.com/FelixKrueger/TrimGalore (with Cutadapt v4.4^28^). Following an initial quality trimming, sequences were trimmed off the 3′ end of the reads if at least 5 bp overlapped with the adapter sequence. Only reads that were no shorter than 35 bp after trimming were kept. Trim Galore was run in non-directional RRBS mode, which performs additional trimming to avoid using cytosine positions in methylation calls that were filled-in during the end-repair step. Low quality bases at the end of the NGS reads were trimmed off (quality trimming using TrimGalore, see RRBS data processing) before the methylation analysis. After read trimming, bisulfite alignment to the GRCh38 (hg38) reference sequence and methylation calling were performed using Bismark v0.24.1^29^ with bowtie2 v2.3.5.1^30^. After mapping, sam files were sorted using samtools v1.10^31^.

To investigate methylation level differences between ALS and Control samples, DMSs (differentially methylated sites, per CpG) and DMRs (differentially methylated regions, per 1000 bp window) were identified using the MethylKit v1.26.0^32^ software package (with R v4.3.1. Both methylated and unmethylated CpG cytosines were imported as an input. Then, sites with fewer than 10 mapped reads and sites with exceptionally high coverage above the 99.9 percentile were excluded from the analysis. To ensure comparability between samples, the CpG methylation data were normalised, setting the median coverage to be the same for each sample (Table 2).

**Table 2:** Methylation statistics of CpG sites.

Sample	N	Min	Median	Mean	Max
ALS61	1622462	0	31.03	45.52	100
ALS69	576758	0	35	46.18	100
ALS74	620129	0	37.04	46.69	100
ALS75	1530019	0	0	28.98	100
ALS76	1272746	0	51.28	49.34	100
ALS81	1444033	0	48	48.71	100
K039	288927	0	50	49.06	100
K107	1465401	0	63.64	51.84	100
K128	428690	0	20	43.07	100
K161	1714994	0	64.71	51.67	100
K190	762914	0	71.43	53.08	100
K257	527931	0	36	46.64	100

**Fig. 3:**
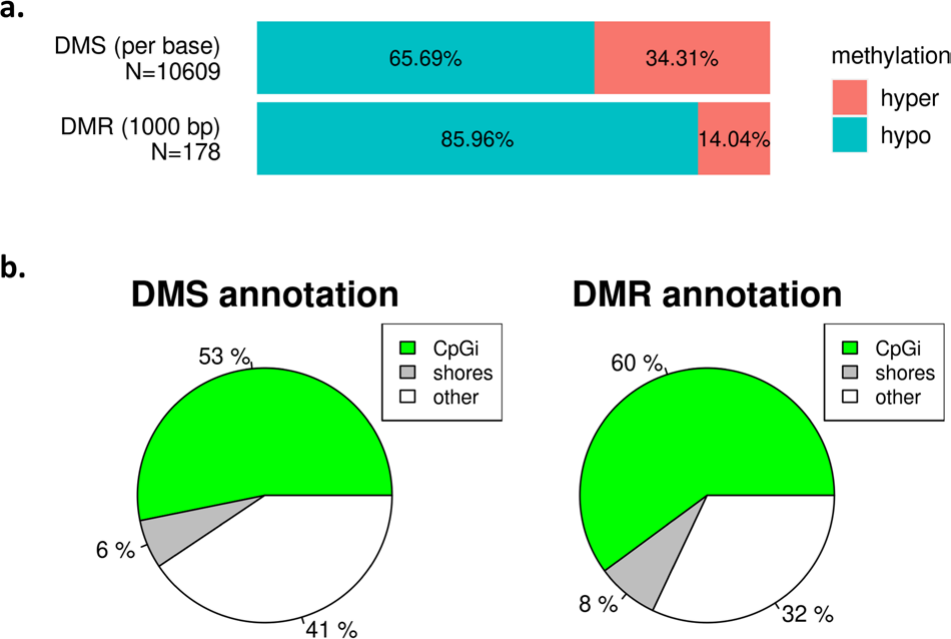
Most DNA methylation occurs in CpG islands and in sequences up to 2 kb distant termed CpG island shores. (**a**) Proportion of hypo- and hypermethylated sites (DMSs) and regions (DMRs) in ALS samples relative to healthy controls. The dominant change is hypomethylation. N: Total number of DMSs and DMRs. (**b**) Annotation of DMSs and DMRs to CpG islands (CpGi) and CpG shores of the human genome.

For differentially methylated site (DMS) analysis, each CpG site’s DNA methylation status was evaluated individually^33^. A CpG site was retained for further analysis only if it had been sequenced and covered in at least 4 samples within each sample group. For the analysis of differentially methylated regions (DMRs), the genome was partitioned into 1000 bp tiling windows with a 1000 bp step-size, and DNA methylation levels were computed for each tiled region. A region was considered for analysis only if it had been sequenced and covered in at least 4 samples within each sample group, and it had a minimum of 5 covered CpG bases. DMS and DMR statistics were calculated by logistic regression using the ‘calculateDiffMeth’ function with default parameters. The threshold for significant methylation differences between sample groups was set to 20%, and the hits were further filtered by a significance value of q <= 0.01. The identified DMSs and DMRs were annotated by the overlapping genic regions, including promoter, exon, intron, and transcription start site (TSS). DMSs and DMRs resulted in extensive coverage of CpG islands (53–60%) and in sequences up to 2 kb distant termed CpG island shores (6–8%)^34^, while also maintaining substantial coverage of other genomic elements (32–41%) (Fig. 3). Description of all software parameter settings employed in the RRBS analysis, reference data and processed data utilized in the analysis can be found in the Data Records section.

### DRIP-seq

**Fig. 4:**
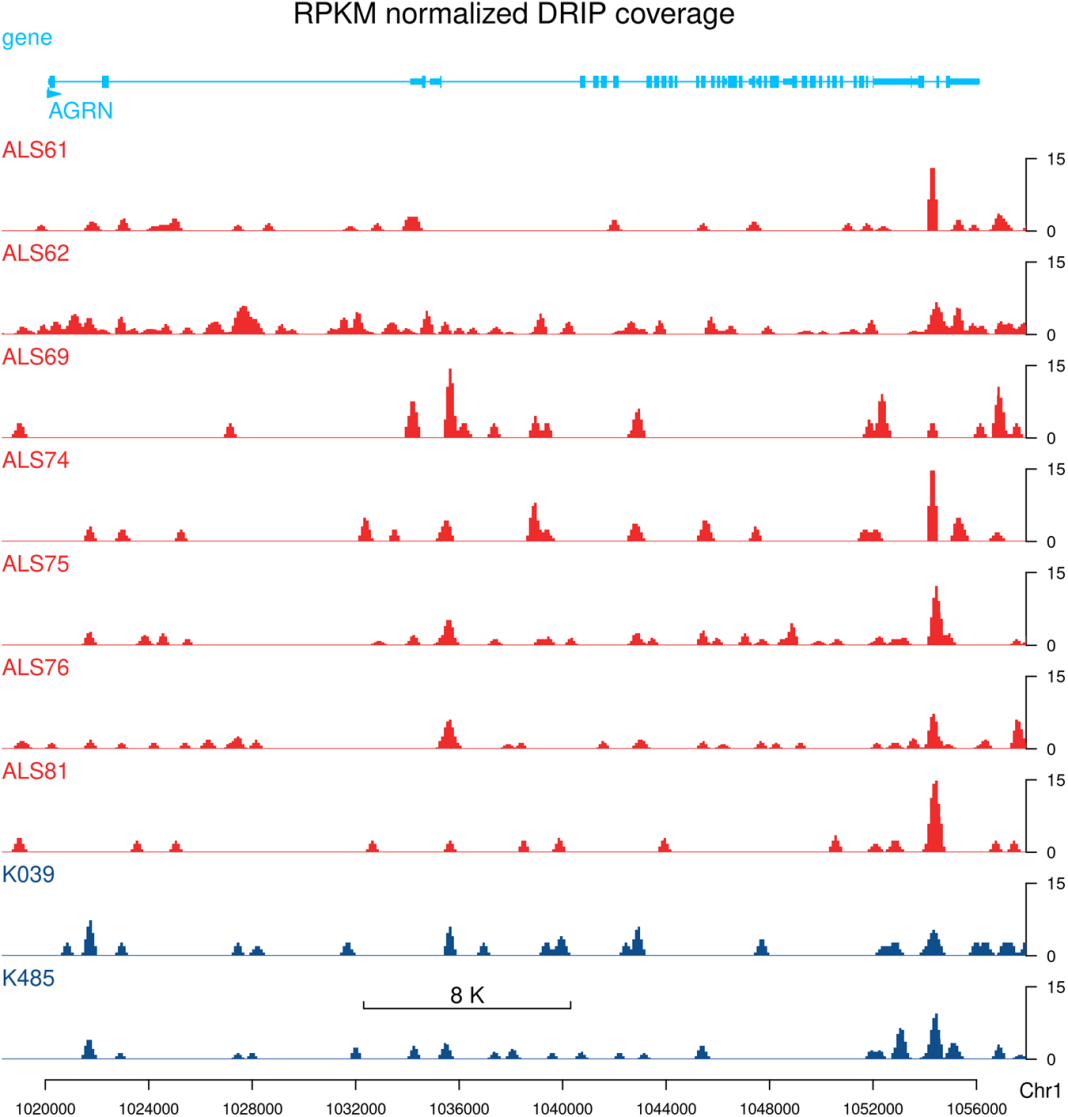
Representative genome browser track showing the RNA-DNA hybrid (R-loop) profile of ALS patients and healthy controls.

DNA-RNA Immunoprecipitation (DRIP) sequencing was started by blocking and coating magnetic beads (Dynal protein G). 2 × 50 μL of protein G beads were washed with 3 × 1 ml of 5 mg/ml PBS/1 mM EDTA/1% BSA solution using a MagnaRack. To the beads, we added 500 µl of 5 mg/ml PBS/1 mM EDTA/1% BSA solution and 25 μg of the RNA-DNA hybrid-specific S9.6 monoclonal antibody^35,36^. The mixture was incubated for 4 hours with rotation at 4 °C in a cold room and then washed with 1 ml of PBS/1 mM EDTA/1% BSA twice. For immunoprecipitation, we diluted each sample to 700 µl with ChIP lysis Buffer (50 mM Hepes/KOH pH 7,5, 0,14 M NaCl, 5 mM EDTA, 1% Triton X-100, 0,1% Na-Deoxycholate). We mixed 200 μL of the sample with 500 μL of ChIP lysis Buffer and added 2 × 700 µl of this mixture to 50 µl of S9.6-coated beads. After incubating overnight at 4 °C with rotation, we performed a series of washing steps using different buffers as described^22^. Finally, the DNA was eluted from the beads by adding 100 µl of IP Elution buffer (50 mM Tris/HCl pH 8.0, 10 mM EDTA, 1% SDS), vortexing every 2 minutes, and incubating in a thermomixer for 15 minutes at 65 °C. We then used a MagnaRack to separate the supernatant and pipetted it into a new low-binding tube. To further purify the DNA, we conducted a PCR clean-up using the NTB M&N kit, eluting the DNA in 3 × 100 μL of H2O (pH > 7). Before NGS library preparation, we performed further fragmentation through sonication in a 1.5 ml low-bind tube (300 μl sample) using a Bioruptor (Diagenode) with 2 × 4 cycles of 30 seconds ON/OFF at LOW settings. Finally, we concentrated the samples to 30 μL using a speed vacuum concentrator and measured the concentration using the Qubit dsDNA HS Assay Kit. NGS library preparation and sequencing was performed as described^36,37^ using the Illumina TruSeq ChIP Sample Preparation protocol. Briefly, DRIP DNA was end-repaired and indexed adapters were ligated to the inserts. Purified ligation products were then amplified by PCR. Amplified libraries were sequenced (at the EMBL Genomics Core Facility, Heidelberg) using an Illumina HiSeq 2500 instrument in the 2 × 125 paired-end sequencing mode. Quality of raw DRIP-seq reads were checked using FastQC v0.11.9 and the results were summarized using MultiQC v1.14^38^. DRIP-seq reads were aligned to the GRCh38 (hg38) reference sequence using bowtie2 v2.3.5.1^30^. Alignments were filtered using samtools v1.10 keeping only primary alignments of properly paired reads that had >30 mapping quality score. PCR and optical duplicates were also filtered out. RPKM normalized coverage (bigwig) files have been generated with 100 bp resolution using bamCoverage v3.5.1^39^. A representative genome browser track is shown in Fig. 4. Detailed software parameter settings can be found in the Data Records section.

### Reference data information

All reference data and databases used for CES, RRBS, and DRIP-seq analyses are listed in Table 3.

**Table 3:** Reference data and reference databases used in this study.

Data description	ID/version	Data processing step
Reference sequence	GRCh38/hg38 (fasta)	read mapping (CES, DRIP, RRBS)
Annotation	GRCh38/hg38, build: p13 (gff3)	gene annotation (CES, RRBS)
Single Nucleotide Polymorphism Database	NCBI dbSNP database	variant ID (CES)
Genome Aggregation Database	gnomAD v2.1.1 (GRCh38 LiftOver)	variant annotation (CES)
Variant database (clinical)	NCBI ClinVar (2022.11.13)	variant annotation (CES)

## Data Records

All NGS datasets generated in this study (CES, RRBS, DRIP-seq) were deposited in Gene Expression Omnibus (GEO) under the accession number: GSE242475^40^. A detailed summary of all NGS experiments, NGS data, processed data, result tables, and identifiers to access relevant datasets for RRBS, CES and DRIP-seq can be found in Tables 4–6.

**Table 4:** Summary of RRBS data generated in this study.

Data description	Tissue	Sample group	Sample ID	SRA/GEO reference	Data collection/Analytical step
RRBS (fastq)	blood	ALS	ALS61	SRX21649976	Reduced representation bisulfite sequencing, 1 × 50 bp reads
	blood	ALS	ALS69	SRX21649977	
	blood	ALS	ALS74	SRX21649978	
	blood	ALS	ALS75	SRX21649979	
	blood	ALS	ALS76	SRX21649980	
	blood	ALS	ALS81	SRX21649981	
	blood	Ctrl	K061	SRX21649982	
	blood	Ctrl	K039	SRX21649983	
	blood	Ctrl	K107	SRX21649984	
	blood	Ctrl	K128	SRX21649985	
	blood	Ctrl	K190	SRX21649986	
	blood	Ctrl	K257	SRX21649987	
CpG methylation data (txt)	blood	ALS	ALS61	GSM7764386	RRBS trimmed read mapping, methylation calling with quality and coverage filtering
	blood	ALS	ALS69	GSM7764387	
	blood	ALS	ALS74	GSM7764388	
	blood	ALS	ALS75	GSM7764389	
	blood	ALS	ALS76	GSM7764390	
	blood	ALS	ALS81	GSM7764391	
	blood	Ctrl	K061	GSM7764392	
	blood	Ctrl	K039	GSM7764393	
	blood	Ctrl	K107	GSM7764394	
	blood	Ctrl	K128	GSM7764395	
	blood	Ctrl	K190	GSM7764396	
	blood	Ctrl	K257	GSM7764397	
Differentially methylated CpG sites (tsv)	blood	ALS vs Ctrl	—	GSE242474	Coverage filtering and normalization, differential methylation analysis, gene annotation, resolution: per CpG site
Differentially methylated regions, 1000 bp (tsv)	blood	ALS vs Ctrl	—	GSE242474	Coverage filtering and normalization, differential methylation analysis, gene annotation, resolution: 1000 bp tiles

**Table 5:** Summary of CES data generated in this study.

Data description	Tissue	Sample group	Sample ID	SRA/GEO reference	Data collection/Analytical step
CES (fastq)	blood	ALS	ALS61	SRX21649845	Clinical exome sequencing, 2 × 150 bp paired-end reads
	blood	ALS	ALS62	SRX21649846	
	blood	ALS	ALS69	SRX21649847	
	blood	ALS	ALS74	SRX21649848	
	blood	ALS	ALS75	SRX21649849	
	blood	ALS	ALS76	SRX21649850	
	blood	ALS	ALS81	SRX21649851	
Clinical exome, gene sequence variations (vcf)	blood	ALS	ALS61	GSM7764370	CES read alignment, sequence variant analysis, annotation
	blood	ALS	ALS62	GSM7764371	
	blood	ALS	ALS69	GSM7764372	
	blood	ALS	ALS74	GSM7764373	
	blood	ALS	ALS75	GSM7764374	
	blood	ALS	ALS76	GSM7764375	
	blood	ALS	ALS81	GSM7764376	
Variant list (tsv)	blood	ALS	—	GSE242472	Merged and reformatted results

**Table 6:** Summary of DRIP-seq data generated in this study.

Data description	Tissue	Sample group	Sample ID	SRA/GEO reference	Data collection/Analytical step
DRIP-seq (fastq)	blood	ALS	ALS61	SRX21651396	DRIP-sequencing, 2 × 125 bp paired-end reads
	blood	ALS	ALS62	SRX21651397	
	blood	ALS	ALS69	SRX21651398	
	blood	ALS	ALS74	SRX21651399	
	blood	ALS	ALS75	SRX21651400	
	blood	ALS	ALS76	SRX21651401	
	blood	ALS	ALS81	SRX21651402	
	blood	Ctrl	K039	SRX21651403	
	blood	Ctrl	K485	SRX21651404	
DRIP coverage (bw)	blood	ALS	ALS61	GSM7764377	DRIP-seq read alignment; quality and duplicate filtering; RPKM-normalized coverage
	blood	ALS	ALS62	GSM7764378	
	blood	ALS	ALS69	GSM7764379	
	blood	ALS	ALS74	GSM7764380	
	blood	ALS	ALS75	GSM7764381	
	blood	ALS	ALS76	GSM7764382	
	blood	ALS	ALS81	GSM7764383	
	blood	Ctrl	K039	GSM7764384	
	blood	Ctrl	K485	GSM7764385	

## Technical Validation

**Fig. 5:**
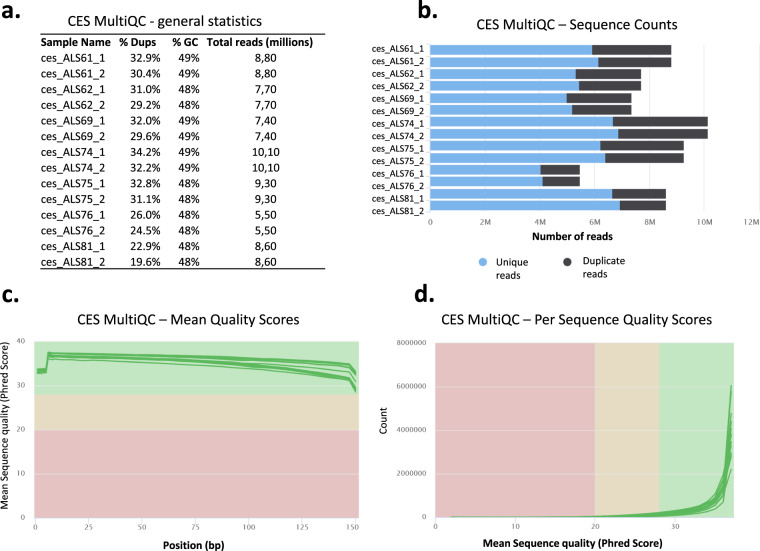
MultiQC validation of clinical exome seq (CES) data. (**a**) General NGS statistics of CES samples. (**b**) The Sequence Counts plot displays the total count of reads, categorized as either unique or duplicate. Identifying duplicates involves requiring an exact sequence match across the entire sequence length. To conduct this analysis, any reads longer than 75 bp were shortened to 50 bp. (**c**) Mean quality Phred scores are presented, with higher scores indicating better base calls. The graph’s background color partitions the y-axis into regions denoting very good quality calls (green zone), calls of moderate quality (orange zone), and calls of poor quality (red zone). (**d**) Per sequence quality Phred scores are depicted, indicating whether a subset of sequences exhibits consistently low-quality values. The background color of the graph distinguishes very good quality calls (green), moderate quality calls (orange), and poor quality calls (red).

CES was validated by FastQC v0.11.9 and summarized reports were generated using MultiQC v1.14^38^. Of the 7.4–10,1 million sequenced reads, greater than 60% of NGS reads were unique (Fig. 5a,b). Mean Phred quality scores for each read position in each RRBS sample were high (indicative of ‘very good quality’) (Fig. 5c). Per sequence Phred quality scores were higher than 28 (indicative of ‘very good quality’) for at least 95% of reads in each CES sample (Fig. 5d).

**Fig. 6:**
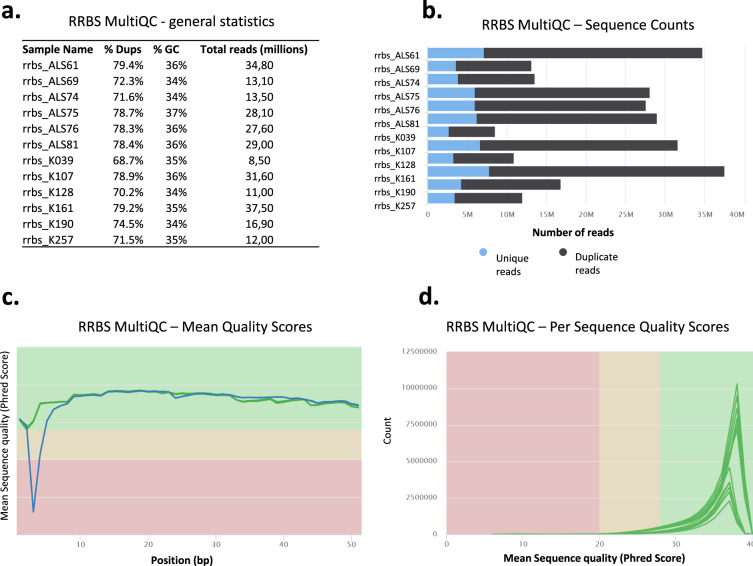
MultiQC validation of reduced representation bisulfite sequencing (RRBS) data. (**a**) General NGS statistics of RRBS samples. (**b**–**d**) Same as in Fig. 2.

In the RRBS setting, 8.5–37,0 million of sequenced reads were obtained (Fig. 6a). Duplication detection indicated 20–30% of NGS reads as unique (Fig. 6b). Mean Phred quality scores for each read position in each RRBS sample were higher than 28 (indicative of ‘very good quality’) (Fig. 6c). Per sequence Phred quality scores were higher than 28 (indicative of ‘very good quality’) for at least 95% of reads in each RRBS sample (Fig. 6d).

**Fig. 7:**
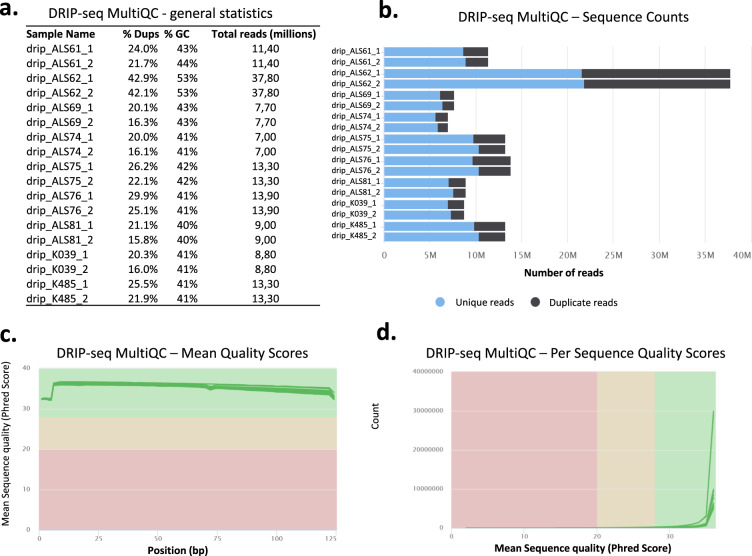
MultiQC validation of DNA-RNA hybrid immunoprecipitation (DRIP) sequencing data. (**a**) General NGS statistics of DRIP-seq samples. (**b**–**d**) Same as in Fig. 2.

For the DRIP-seq experiments, raw sequence coverage ranged from 7.0–37.7 million reads (Fig. 7a), of which greater than 70 percent qualified as unique (Fig. 7b). Mean and Per sequence Phred quality scores for each read position in each DRIP-seq sample were high (indicative of ‘very good quality’) (Fig. 7c,d).

## Usage Notes

**Fig. 8:**
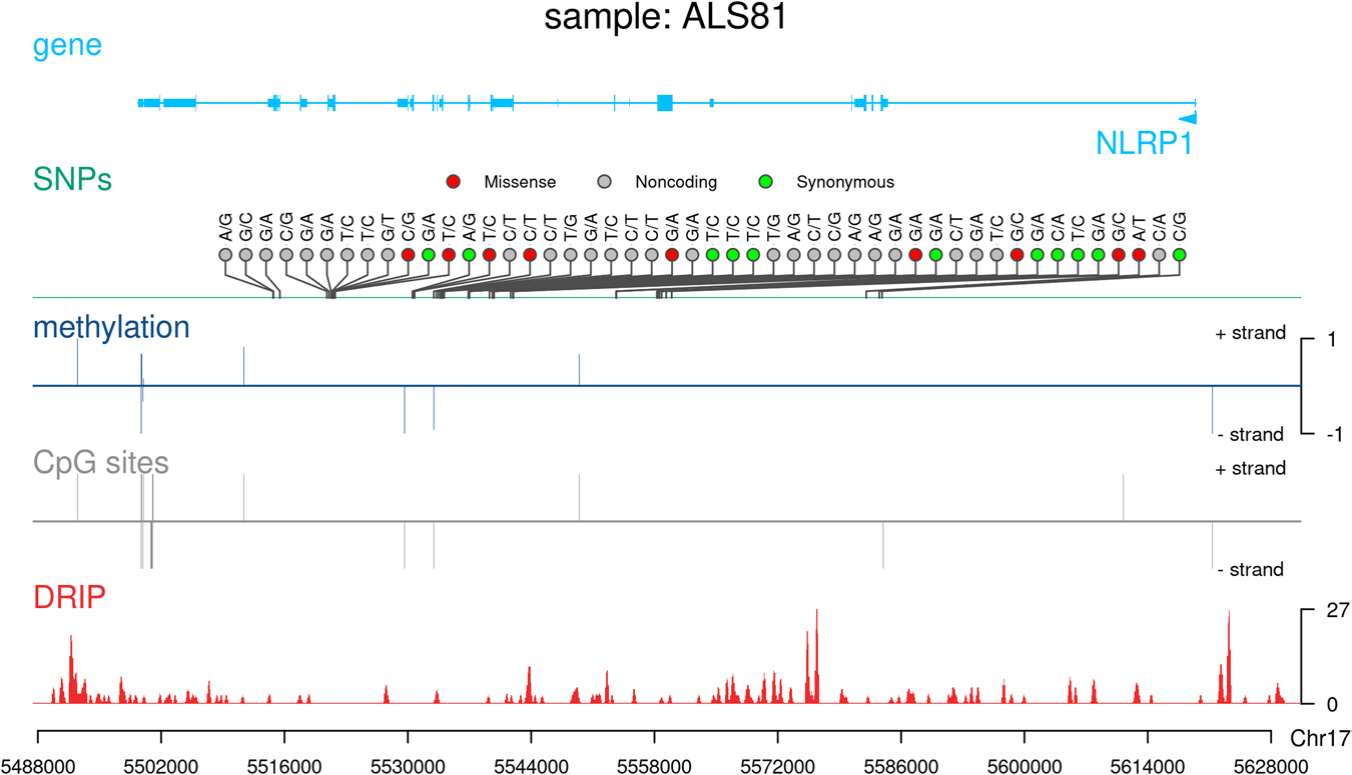
Correlative analysis of CES, RRBS, and DRIP profiles in ALS patients. Lolliplots show the mutational spectra (SNPs) of the displayed gene (Reference base/Alternate base). Missense, synonymous, and noncoding variants are shown in different colours. DNA methylation is represented by the ratio of methylated/unmethylated CpG dinucleotides. + values: fw strand; - values: reverse strand. The position of CpG sites are shown as a proxy. DRIP stands for the RPKM normalized read coverage of RNA-DNA hybrid (R-loops) levels over the genomic region.

The NGS results presented above reveal three distinct data layers in a small group of ALS patients, encompassing genetic changes, DNA methylation alterations, and R-loop modifications. Obtaining such data from individual patients is crucial as it enables the correlation of these variables both within and between individuals (Fig. 8). Such a correlative analysis is expected to unveil significant epigenetic patterns, potentially explaining the missing heritability (Mendelian inheritance) of gene mutations identified in ALS. The data presented here have been established using widely recognized software tools, making them readily adaptable for integration into any genome analysis workflow. We are confident that the data we provide can contribute to better understand the aetiology of this devastating disease .

**Table 7:** Summary of software tools used in the current study.

Software version	Data processing step	Parameter settings
TrimGalore v0.6.10; cutadapt v4.4	Read trimming (RRBS)	–quality 20–stringency 5–length 35–non_directional–rrbs
Bismark v0.24.1; bowtie2 v2.3.5.1	Bisulfite alignment (RRBS)	reference sequence: GRCh38; -N 1
samtools v1.10	Alignment sorting (RRBS)	—
MethylKit v1.26.0; R v4.3.1	Methylation calling (RRBS)	processBismarkAln(): read.context = CpG, mincov = 10
	Methylation data filtering and normalization (RRBS)	filterByCoverage(): lo.count = 10, hi.perc = 99.9; normalizeCoverage(): method = “median”
	DMS analysis (RRBS)	unite(): min.per.group = 4 L, destrand = TRUE; calculateDiffMeth(): default parameters; getMethylDiff(): difference = 20, qvalue = 0.01
	DMR analysis (RRBS)	tileMethylCounts(): win.size = 1000, step.size = 1000, cov.bases = 5; unite(): min.per.group = 4 L; calculateDiffMeth(): default parameters; getMethylDiff(): difference = 20, qvalue = 0.01
NextGENe 2.4.2.3	Read alignment (CES)	reference sequence: GRCh38
	Variant calling (CES)	>5 reads/variant position; >20% of reads present the variant
	Variant annotation (CES)	databases: NCBI dbSNP; gnomAD v2.1.1 (GRCh38 LiftOver); NCBI ClinVar (2022.11.13)
	Gene annotation (CES)	annotation database: GRCh38.p13
R v4.3.0	Data formatting (CES)	—
bowtie2 v2.3.5.1	Read alignment (DRIP)	reference sequence: GRCh38
samtools v1.10	Alignment filtering (DRIP)	view -q30 -f3 -F3840
bamCoverage v3.5.1	Normalized coverage (DRIP)	–normalizeUsing RPKM–binSize 100–smoothLength 300

## Data Availability

No custom code was generated or applied for analysis of the genomic data presented. All software tools used for the analyses and the applied parameter settings are detailed in Table 7.
